# Cytotoxicity, Antiadipogenic, Low-Density Lipoprotein Oxidation Inhibitory Activities, and Acute Toxicity Study of *Psychotria densinervia* Hydroethanolic Leaf and Bark Extracts

**DOI:** 10.1155/tswj/1732653

**Published:** 2024-12-17

**Authors:** Jean Romuald Mba, Djamila Zouheira, Stephanie Tamdem Guetchueng, Hadidjatou Daïrou, Paul Toukam Djouonzo, Lawrence Ayong, Jules-Roger Kuiate, Gabriel A. Agbor

**Affiliations:** ^1^Centre for Research on Medicinal Plants and Traditional Medicine, Institute of Medical Research and Medicinal Plants Studies, P.O. Box 13033, Yaoundé, Cameroon; ^2^Department of Biochemistry, Faculty of Sciences, University of Dschang, P.O. Box 67, Dschang, Cameroon; ^3^Malaria Research Unit, Centre Pasteur de Yaounde, P.O. Box 1274, Yaoundé, Cameroon

**Keywords:** antiadipogenic, low-density lipoprotein, oxidation, *Psychotria densinervia*, SW-872 cells, toxicity

## Abstract

**Background:** Obesity is increasingly taking an important stage as a cause of death worldwide, and interventions with a good cost-effectiveness ratio are needed. *Psychotria densinervia* is one of these natural products with health benefits. Objective. The present study evaluated the cytotoxicity, antiadipogenic, low-density lipoprotein (LDL), oxidation inhibitory activities, and acute toxicity of *Psychotria densinervia* hydroethanolic leaf and bark extracts.

**Methods:** The cytotoxicity evaluation of the extracts (62.5, 125, 250, and 500 μg/mL) using the MTT assay and the antiadipogenic activity (25, 50, 100, and 200 μg/mL) using oleic acid were carried out in SW-872 cells. Copper sulfate (CuSO_4_)-induced oxidation was used in the evaluation of the effect of extracts (0.25, 0.5, and 1 mg/mL) against LDL oxidation. The oral acute toxicity evaluation of a single dose of 2000 mg/kg of the extracts was performed in Wistar albino rats weighing 127 ± 2 g.

**Results:** The leaf and bark extracts did not show any sign of cytotoxicity at the tested concentrations. The best antiadipogenic activity was observed by the standard orlistat (38.45 ± 1.70 μg/mL), followed by the leaf extract (IC_50_: 41.47 ± 0.50 μg/mL) and the least the bark extract (IC_50_: 107.50 ± 0.90 μg/mL). At a concentration of 1 mg/mL, the leaf extract presented an oxidation lag time of 130 min, which was higher and better than that of the bark extract (120 min). Quercetin (standard) presented an oxidation lag time longer than 3 h. The oral acute toxicity evaluation did not show any signs of toxicity indicating that the LD_50_ was greater than 2000 mg/kg.

**Conclusion:** Based on the results obtained, the *P. densinervia* hydroethanolic leaf extract possesses a better antioxidant and antiadipogenic activities than the bark extract.

## 1. Introduction

Obesity remains a global health problem associated with an increase in several risk factors responsible for the development of many chronic and metabolic disorders. These disorders include hypertension, fatty liver disease, dyslipidemia, type 2 diabetes, osteoarthritis, obstructive sleep apnea, gallstones, and atherosclerosis [1]. Over the years, the number of obese subjects has been on the rise and is predicted to triple by 2030 [2]. The latest statistics show that 18.4% of women and 7.8% of men in Africa live with obesity. In 2019, 24% of the world's overweight children aged under five lived in Africa [3]. The prevalence of obesity in the adult Cameroonian population increased steadily from 5.8% in 2000 to 11.4% in 2016 [4]. These reports highlight the fact that the obesity epidemic is increasingly taking an important place as a cause of disease worldwide and may continue to deteriorate if nothing is done. Orlistat, a lipase inhibitor that blocks the breakdown of triglycerides, is a contemporary synthetic antiobesity medicine in use today [5]. Though efficient, some complications such as respiratory infection, nephrotoxicity, oily stools, dyspepsia, flatulence, and abdominal pain, as well as menstrual disorders, have been linked with this Orlistat [6, 7]. Hence, it is important to look for new targets and new alternatives as antiobesity agents. Excessive food and high energy intake have been reported to play a central role as causative factors of obesity through adipogenesis (increased numbers of fat cells) and expansion of adipose tissues [8, 9] and size hypertrophy of individual adipocytes [10]. Another trigger of obesity is oxidative stress, which increases the accumulation of white fat and alters food intake [7, 11]. Earlier studies had reported a positive and significant correlation between adipose tissue accumulation and circulating levels of oxidized LDL (ox-LDL) [12–14]. Similarly, higher ox-LDL concentrations are linked with a high level of triglycerides and a low level of HDL cholesterol [15, 16]. Thus, the inhibition of adipogenesis and LDL oxidation may be an important step toward the management of obesity.

Complementary and alternative medicine plays a significant role in the management of obesity and overweight in many countries worldwide. However, the adoption of this medicine by populations often poses a certain number of problems because there is little scientific data on safety and effectiveness of the products of this medicine. Among medicinal plants, species from the Rubiaceae family are some of the most extensively studied, with hundreds of reports in the scientific literature concerning their diverse pharmacological effects and, remarkably, their therapeutical activities related to metabolic syndrome and associated chronic noncommunicable diseases, such as antioxidant, anti-inflammatory, metabolic regulatory, antiadipogenic, hypoglycemic, antihypertensive, and hypolipidemic effects [17, 18]. In this article, an emphasis has been placed on the study of *Psychotria densinervia*, a Rubiaceae with little study on its biological activities. In traditional medicine, *P. densinervia* is frequently used for the treatment of malaria. Additionally, it would aid in digestion and have diuretic qualities, earning it the nickname “slimming plant” in the southern region of Cameroon. Our previous research reported the in vitro antioxidant, anti-inflammatory, and digestive enzyme inhibitory properties of *P. densinervia* [19]. The present study evaluated the cytotoxicity, antiadipogenic, low-density lipoprotein (LDL) oxidation inhibitory activities, and acute toxicity study of *P. densinervia* hydroethanolic leaf and bark extracts.

## 2. Materials and Methods

### 2.1. Chemicals

Dulbecco's modified Eagle's medium (DMEM), fetal bovine serum (FBS), penicillin, streptomycin, bovine serum albumin (BSA), oleic acid, PBS, formalin, isopropanol, MTT assay kit, lipid (Oil Red O) staining kit, dimethyl sulfoxide (DMSO), ketamine, solid potassium bromide (KBr), sodium chloride (NaCl), ethylenediaminetetra acetic acid (EDTA), copper sulfate (CuSO_4_), quercetin, trichloroacetic acid (TCA), thiobarbituric acid (TBA), sodium hydroxide (NaOH), and Bradford Protein Assay Kit were purchased from Sigma-Aldrich, Co. (St. Louis, MO, USA).

### 2.2. Plant Material

*Psychotria densinervia* fresh leaf and bark were collected in a village called “Meyomessala,” located in the southern region of Cameroon, in October 2022, at around 7 a.m. The specimen was identified at the National Herbarium of Yaoundé, Cameroon, by an Ethnobotanist Dr. Tsabang Nole using the identification number 58226 HNC. The bark and leaf of *P. densinervia* were shade-dried for 2 weeks at room temperature after being cleaned three times with distilled water [19].

### 2.3. Preparation of *Psychotria densinervia* Hydroethanolic Leaf and Bark Extracts

The process previously outlined was used to prepare the hydroethanolic leaf and bark extracts of *P. densinervia* [19]. After grinding, *P. densinervia* leaf (546.2 g) and bark (426.6 g) were macerated separately in a hydroethanolic solution (70% ethanol and 30% distilled water, v/v) at 35°C using a closed percolator for 72 h. The extracts were filtered, evaporated with rotavapor, and then oven-dried at 50°C for 48 h. The leaf and bark extracts were labeled and stored at −4°C until needed [19].

### 2.4. Cell Viability Assay

The human liposarcoma SW-872 cell line obtained from the American Type Culture Collection (ATCC) was used in this paper. The cytotoxic effect of hydroethanolic leaf and bark extracts was determined in MTT assay [20]. In 96-well plates, cells were seeded at a density of 1 × 10^4^ cells/well. After 24 h, they were treated with plant extracts at different concentrations (62.5, 125, 250, and 500 μg/mL) for 3 days at 37°C in 5% CO_2_ humidified air. 20 μL of filter-sterilized MTT (2 mg/mL) in phosphate-buffered saline (PBS) was added to each well and incubated at 37°C for 3 h. The medium with MTT was removed, and the formed formazan crystals were solubilized by the addition of 100 μL of DMSO, and the absorbance was read at 540 nm using a universal microplate reader. The percentage of viable cells was obtained by dividing the mean absorbance of treated cells (for each concentration of extract) by the mean absorbance of the untreated cells.

(1) $$\begin{array}{c} \text{Percentage of cell viability}=\left(\frac{\text{mean absorbance of extracts treated cells}}{\text{mean absorbance of untreated cells}}\right)\times 100. \end{array}$$

### 2.5. Evaluation of Antiadipogenic Effect of *Psychotria densinervia* Hydroethanolic Leaf and Bark Extracts

#### 2.5.1. Evaluation of the Effects of *Psychotria densinervia* Hydroethanolic Leaf and Bark Extracts on SW-872 Differentiation

In 24-well microliter plates, human SW-872 liposarcoma fibroblast obtained from ATCC was cultured in DMEM containing 10% FBS, 100 U/mL penicillin, and 100 μg/mL streptomycin at 37°C in 5% CO_2_ until confluence was reached (100% confluence) and growth was arrested. It is at this point that they begin differentiating into adipocytes. The medium was replaced every 3 days. The impact of *P. densinervia* extracts on the development of SW-872 preadipocytes into mature adipocytes was assessed using the Dordevic, Konstantopoulos, and Cameron-Smith [21] method of lipid uptake inhibition. Previous cell cultures were maintained in DMEM/Ham's F-12 medium supplemented with 0.6 mol/L oleic acid (a differentiation agent) and 1% BSA. Hydroethanolic leaf and bark extracts of *P. densinervia* were applied to the corresponding test wells at varying concentrations (25, 50, 100, and 200 μg/mL). Orlistat was used as positive control at the same concentrations.

The SW-872-differentiated cells that had only received oleic acid treatment were used as the negative control, and the SW-872 undifferentiated cells that did not receive oil acid treatment were used as the normal control. Cells were cultured for 3 days until 90% of confluences were reached.

#### 2.5.2. Staining of Lipid Droplets

On day 3, Oil Red O staining was done using a modified approach by Ramírez-Zacarías, Castro-Muñozledo, and Kuri-Harcuch [22]. After being cleaned with PBS, SW872 adipocyte cells were fixed with 10% formalin. After three rounds of deionized water rinsing, fixed cells were stained for 30 minutes at room temperature using a working solution of Oil Red O stain (0.5% in isopropanol). After that, a phase-contrast microscope equipped with a 200-X magnification digital camera was used to take a picture of this. After dissolving the lipid droplets with isopropanol, the absorbance was measured at 492 nm. The relative lipid content and adipogenesis inhibitory percentage were calculated as follows:

(2) $$\begin{array}{c} \text{relative lipid content }\%=\left(\frac{\text{absorbance of sample}}{\text{absorbance of negative control}}\right)*100,\\ p\text{ercentage of adipogenesis inhibiton}=\left(\frac{\text{absorbance of negative control }–\text{absorbance of sample}}{\text{absorbance of negative control}}\right)*100. \end{array}$$

### 2.6. Evaluation of the Effects of *Psychotria densinervia* Hydroethanolic Leaf and Bark Extracts on LDL Oxidation Caused by CuSO4

#### 2.6.1. Evaluation of the Formation of Conjugated Dienes

The isolated human LDL was purchased from Biogenuix Medsystems Pvt. Ltd., India (Lot No. 819PLDL07). Using 10 mM of PBS (pH = 7.4), the LDL protein concentration (10 mg/mL) was adjusted to 150 μg/mL. One milliliter of different concentrations of aliquot extracts (0.25, 0.5, and 1 mg/mL) was mixed with 1 mL of LDL solution. The addition of 0.1 mL of a newly made 10 μM CuSO_4_ solution at 37°C started the oxidation of LDL [23]. Subsequently, samples with LDL and copper sulfate without extract (the negative control sample) were prepared in the same condition. Quercetin was used as the positive control. Using a UV/visible spectrophotometer, the emergence of conjugated diene was observed at 234 nm once every 10 minutes for 3 hours at 37°C. Next, a plot of the oxidation–time curve was done, and the lag time (induction phase of LDL oxidation) and time 50 (time to obtain 50% of LDL oxidation) were recorded.

#### 2.6.2. Evaluation of Thiobarbituric Acid Reactive Substance (TBARS) Formation

After the CuSO_4_ oxidation process, 0.1 mL of EDTA (2 mM) was added to the sample mixture to end the oxidation process. Afterward, 1 mL of TCA 15%, 1 mL of TBA 0.67%, and 0.05 N of NaOH were added and incubated for an additional 20 min at 90°C in an oven. Following a cooling period to ambient temperature and a 15-min centrifugation at 3000 rpm, the absorbance at 532 nm was measured [23]. The inhibition percentage (%) of TBARS formation was calculated using the following equation:

(3) $$\begin{array}{c} \text{inhibition }\left(\%\right)=\left(\frac{\left(\text{absorbance of the negative control}-\text{absorbance of the reaction mixture}\right)}{\text{absorbance of the negative control}}\;\right)\times 100. \end{array}$$

IC_50_ (concentration of the sample to produce half maximal inhibition) value was calculated from the nonlinear regression graph using GraphPad Prism 5.

### 2.7. Acute Toxicity Assay of *Psychotria densinervia* Hydroethanolic Leaf and Bark Extract

*Psychotria densinervia* extracts' oral acute toxicity was evaluated in accordance with the Organization for Economic Co-operation and Development (OECD) guideline 425 [24]. A total of fifteen female Wistar albino rats, nonpregnant and nulliparous, weighing 127 ± 2 g and 8–12 weeks old, were provided by the animal house of the Institute of Medical Research and Medicinal Plants Studies (IMPM), Yaoundé, Cameroon. For a duration of seven days, the rats were acclimatized in metal enclosures with ambient temperature, a 12-h natural light and dark cycle, and unrestricted access to regular rat food and fresh water. Animal welfare laws and the 2011 Manual for the Treatment and Utilization of Lab Animals, 8th Edition, were respected in the handling of the animals. The study was carried out in compliance with the Joint Institutional Review Board for Animal & Human Bioethics (JIRB) practice and principles on the use of experimental animals (ethical clearance reference no: BTC-JIRB2024-098).

The rats were divided into three groups (I, II, and III), each with five rats. Distilled water (10 mL/kg) was given to group I (the control group), while *P. densinervia* hydroethanolic leaf and bark extracts were given to groups II and III (the test groups) in a single dosage of 2000 mg/kg body weight. After dosage, every animal was examined for 5 min, then every 15 min for 4 h, every 30 min for 6 h, once a day for 48 h, and for up to 14 days in case there was a long-term, potentially fatal consequence. Toxicological symptoms were noted, such as salivation, trembling, convulsions, diarrhea, changes in fur or eyes, breathing difficulties, itching, coma, or death. Water and food intake were recorded every day. The weight of the animals was recorded every 2 days.

### 2.8. UPLC-ESI/MS Analysis

*P. densinervia* leaves and bark hydroethanolic extracts were diluted in HPLC grade methanol (5 mg/mL) and filtered. 5 µL aliquot of each sample was injected into the Ultimate Dionex 3000 UPLC (Germany) connected to a HRESI-QTOF Spectrometer (Bruker, Germany) The spectrometer was set to the positive mode of operation (m/z range: 100–1500, scan rate: 1.00 Hz). The spray voltage was 4.5 kV, the capillary temperature was 200°C, and the sheath gas was nitrogen (10 L/min). HPLC separation was accomplished using a Synergi MAX-RP 100A (50 × 2 mm, 2.5 m particle size) column held at 40°C with H_2_O/acetonitrile acidified with 0.1% HCOOH as a mobile phase. The flow rate was 0.5 mL/min, and the gradient was as follows: H_2_O/acetonitrile 10/90 (0 min), with a curve gradient of 5 until 1.5 min; then, the polarity was gradually increased to 100% of acetonitrile for 8 min, isocratic 0/100 for 0.5 min, and finally re-equilibration to 10/90 for 1 min.

### 2.9. UPLC-ESI/MS Data Analysis and Compounds Annotation

Raw data files from the Bruker spectrometer MS (. d) were converted to a format compatible with our analysis software (. raw to. mzXML). Spectral data (. mzXML files) were visualized in MZmine 2.53. The converted data were processed with MZmine 2.53 [25], using the following workflow: retention time window: 0–10 min; total intensity threshold: 10,000. Alignment was performed with adaptive curve model. Maximum RT shift was 0.01 min, and maximum mass tolerance was 10 ppm. For detecting and grouping unknown compounds, S/N threshold: 15; minimum intensity threshold: 10,000; RT tolerance: 0.1 min; S/N threshold for gap filling: 25. The feature sets obtained, saved as a. mgf file, was exported from MZmine and processed with SIRIUS [26]. The parameters used were Instrument profile: QTOF; mass accuracy: 10 ppm for MS2; possible ionizations: [M + H]^+^, [M + NH4]^+^, [M + K]^+^, [M + Na]^+^, [M − H_2_O + H]^+^, [M + H_2_O + H]^+^; the ZODIAC score threshold was set to 0.8 for formula prediction. Only structures with a SIRIUS score above 80% were considered.

### 2.10. Statistical Analysis

The mean ± SD was used to express the results. GraphPad Prism version 5.00 for Windows (GraphPad software) was used to do the statistical analysis. A one-way ANOVA was employed with a significance level of *p* < 0.05. The IC_50_ values were determined using nonlinear regression. The Pearson correlation between LDL oxidation inhibition versus lipid uptake percentage of inhibition, relative lipid content, and lipid uptake percentage of inhibition versus relative lipid were determined using GraphPad Prism version 5.00 for Windows (GraphPad software).

## 3. Results

### 3.1. Effects of *Psychotria densinervia* Hydroethanolic Leaf and Bark Extracts on Cell Viability

**Figure 1:**
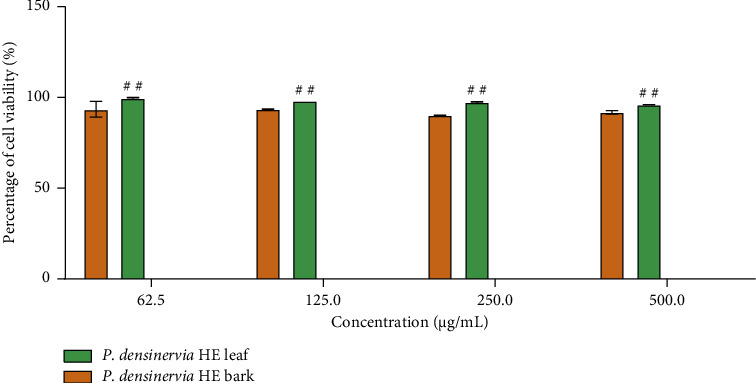
Effects of *Psychotria densinervia* hydroethanolic leaf and bark extracts on SW-872 cell viability using the MTT assay. *P. densinervia* HE = *Psychotria densinervia* hydroethanolic; values are expressed as mean ± SD; *n* = 3; significant difference at ^##^*p* < 0.01.

The effect of *P. densinervia* hydroethanolic leaf and bark extracts on cell viability is presented in Figure 1. Both *P. densinervia* hydroethanolic leaf and bark extracts did not show any significant (*p* > 0.05) toxicity after 3 days of treatment at all tested concentration. The hydroethanolic leaf extract exhibited a cell viability significantly higher (*p* < 0.001) than the bark extract at all concentrations.

### 3.2. Antiadipogenic Effect of *Psychotria densinervia* Hydroethanolic Leaf and Bark Extracts

**Figure 2:**
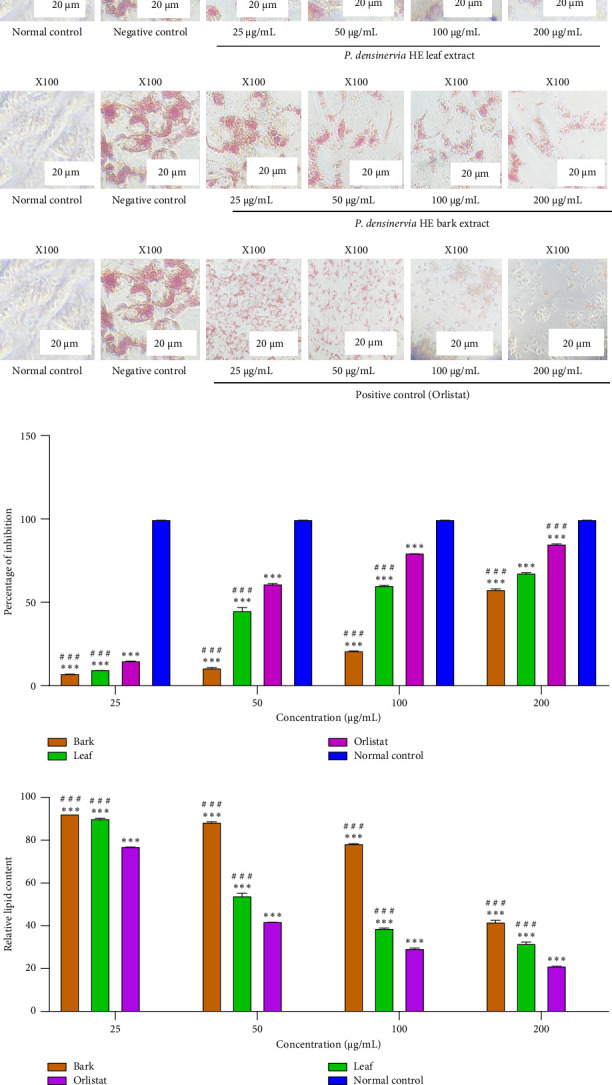
Effects of *Psychotria densinervia* hydroethanolic leaf and bark extracts on SW-872 lipid uptake inhibition. (a) Representative images showing lipid accumulation in cells treated with different concentrations of *Psychotria densinervia* hydroethanolic leaf and bark extracts (cells were observed under magnification of 100X, scale bar, 20 µm); (b) inhibition percentage of lipid accumulation by *Psychotria densinervia* hydroethanolic leaf and bark extracts; (c) relative lipid content in cells treated by *Psychotria densinervia* hydroethanolic leaf and bark extracts. *P. densinervia* HE = *Psychotria densinervia* hydroethanolic; negative control = SW-872 cells + oleic acid treatment (differentiated cells); normal control: SW-872 cells without oleic acid treatment (undifferentiated cells); values are expressed as mean ± SD; *n* = 3; ⁣^∗∗∗^*p* < 0.001: significant difference compared to normal control; ^###^*p* < 0.001: significant difference compared to orlistat (positive control).

The microscopic examination of the Oil Red O staining revealed a concentration-dependent decrease of lipid content in the extract-treated wells compared to the negative control wells (Figure 2(a)). The normal control wells did not exhibit any lipid uptake. The red color observed in differentiated SW-872 adipocytes (negative control) indicates the abundance of lipid (triglyceride) uptake. For that, a concentration-dependent inhibition in lipid uptake was observed with *P. densinervia* hydroethanolic leaf and bark extracts as well as the standard orlistat. The percentages of inhibition and relative lipid content (Figures 2(b) and 2(c)) obtained are in accordance with the microscopic observations. At a concentration of 200 μg/mL, orlistat exhibited a percentage of inhibition and a relative lipid content of 85.25% ± 0.5% and 21.40% ± 0.43%, respectively, followed by the leaf extract (67.82% ± 0.96% and 31.93% ± 1.18%, respectively) and the bark extract (57.88% ± 1.04% and 42.01% ± 1.18%, respectively). The hydroethanolic leaf extract exhibited an IC_50_ value of 41.47 ± 0.50 μg/mL, which was significantly (*p* < 0.001) lower than that of the bark extract (IC_50_: 107.50 ± 0.90 μg/mL). Orlistat exhibited an IC_50_ value of 38.45 ± 1.70 μg/mL (Table 1).

### 3.3. Effect of *Psychotria densinervia* Hydroethanolic Leaf and Bark Extracts on Low-Density Lipoprotein Oxidation Induced by CuSO_4_

#### 3.3.1. Effect of *P. densinervia* Hydroethanolic Leaf and Bark Extracts on LDL Oxidation Lag Time and Time 50

**Figure 3:**
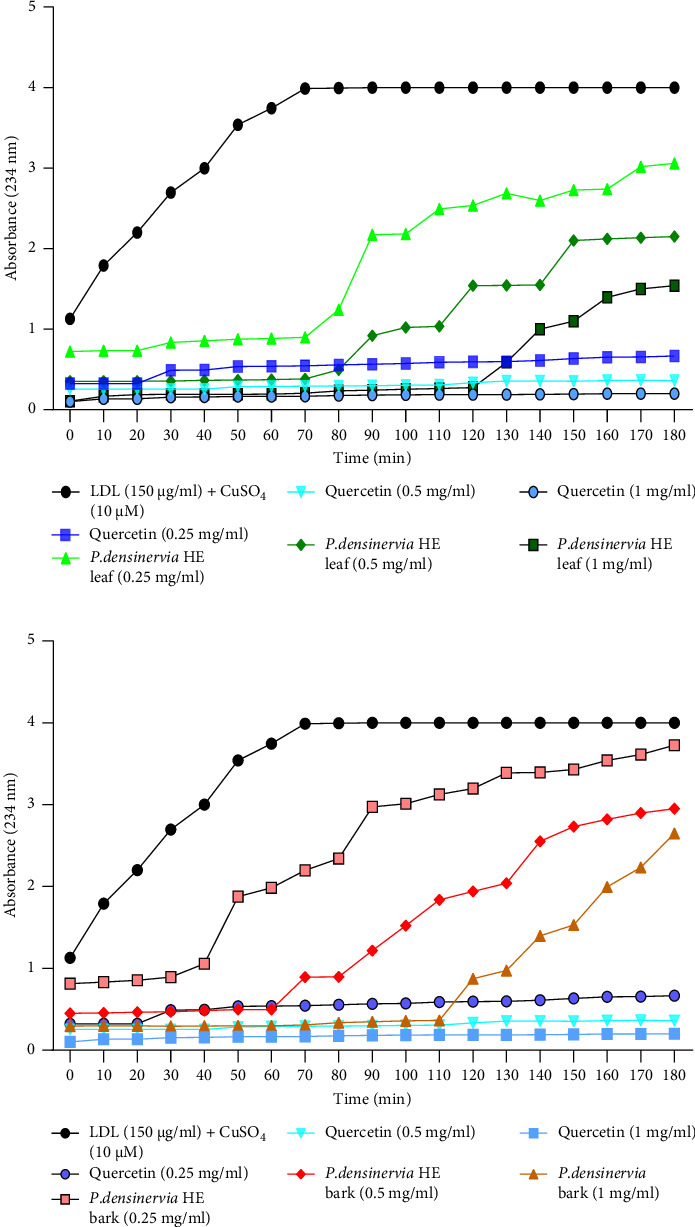
Effect of *Psychotria densinervia* hydroethanolic leaf and bark extracts on LDL oxidation lag time. *Psychotria densinervia* hydroethanolic leaf (a) and bark (b) extracts as well as quercetin were used at the concentrations of 1 mg/mL, 0.5 mg/mL, and 0.25 mg/mL. LDL + CuSO_4_ = low-density lipoprotein + copper sulfate; *P. densinervia* HE = *Psychotria densinervia* hydroethanolic.

The effect of *P. densinervia* hydroethanolic leaf and bark extracts on the LDL oxidation lag time and time 50 is presented in Figure 3. A concentration-dependent increase in LDL oxidation lag time and time 50 were observed with *P. densinervia* hydroethanolic leaf and bark extracts as well as quercetin. At a concentration of 1 mg/mL, *P. densinervia* hydroethanolic leaf extract exhibited a lag time of 130 min (Figure 3(a)). At the same concentration, the bark extract exhibited a lag time of 120 min (Figure 3(b)). At concentrations of 0.5 and 0.25 mg/mL, the lag times were 90 and 80 min, respectively, for the leaf extract (Figure 3(a)) while the bark extract exhibited 70 and 40 min at these concentrations, respectively (Figure 3(b)). In the samples containing quercetin, the optical density increased from 0.32 to 0.66, 0.25 to 0.36, and 0.13 to 0.20 at concentrations of 0.25, 0.5, and 1 mg/mL, respectively, between 0 and 3 h, with lag times equal to or higher than 3 h. At the same time, the time 50 of the LDL oxidation with *P. densinervia* hydroethanolic leaf extract was 110 min at the concentration of 0.25 mg/mL and equal to or higher than 3 h at the concentrations of 0.5 and 1 mg/mL while the *P. densinervia* hydroethanolic bark extract exhibited a time 50 of 80 min at a concentration of 0.25 mg/mL, 140 min at a concentration of 0.5 mg/mL, and 175 min at a concentration of 1 mg/mL. The time 50 exhibited by the standard quercetin was equal to or higher than 3 h at all tested concentrations.

#### 3.3.2. Effect of *Psychotria densinervia* Hydroethanolic Leaf and Bark Extracts on the Formation of TBARS

The effect of *P. densinervia* hydroethanolic leaf and bark extracts on lipid peroxidation end product (TBARS) formation is presented in Table 2. No significant (*p* > 0.05) difference in the inhibition percentage was observed between *P. densinervia* hydroethanolic leaf extract (94.5% ± 0.8%) and quercetin (95.6% ± 0.4%) at a concentration of 1 mg/mL. At a concentration of 0.5 mg/mL, quercetin exhibited an inhibition percentage of 79.9% ± 0.6%, which was greater (*p* < 0.05, *p* < 0.01) than that of *P. densinervia* hydroethanolic leaf extract (66.8% ± 0.8%) and the bark extract (53.2% ± 0.8%). Quercetin also exhibited a significant (*p* < 0.001) higher inhibition percentage (74.0% ± 0.6%) than that of *P. densinervia* hydroethanolic leaf (57.2% ± 0.4%) and bark extract (38.5% ± 1%) at a concentration of 0.25 mg/mL. The best IC50 value was obtained with quercetin (IC50: 0.10 ± 5.10 mg/mL), followed by *P. densinervia* hydroethanolic leaf extract (IC_50_: 0.20 ± 8.50 mg/mL) and at last, hydroethanolic bark extract (IC_50_: 0.24 ± 6.50 mg/mL).

### 3.4. Pearson Correlation Between LDL Oxidation Inhibition Versus Lipid Uptake Percentage of Inhibition, Relative Lipid Content, and Lipid Uptake Percentage of Inhibition Versus Relative Lipid Content

Correlation analysis revealed that there was a positive and significant (*p* < 0.05) correlation between LDL oxidation inhibition versus lipid uptake percentage of inhibition (*r* = 0.89), while there was a negative and significant (*p* < 0.05) correlation between LDL oxidation inhibition versus relative lipid content (*r* = −0.89) (Table 3). The positive correlation recorded between LDL oxidation inhibition versus lipid uptake percentage of inhibition suggest that the inhibition of LDL oxidation is linked to the increase of lipid uptake percentage of inhibition. In addition, the negative correlation recorded between LDL oxidation inhibition versus relative lipid content suggested the link between the increase of LDL oxidation inhibition and the decrease of relative lipid content. In the other hand, a negative and significant (*p* < 0.001) correlation between lipid uptake percentage of inhibition versus relative lipid content (*r* = −1) was also observed as well showing that the more the percentage of inhibition increase, the more the relative lipid content decrease.

### 3.5. Acute Oral Toxicity Effects of *Psychotria densinervia* Hydroethanolic Leaf and Bark Extracts

#### 3.5.1. Physical and Behavioral Signs of Toxicity

The administration of *P. densinervia* hydroethanolic leaf and bark extracts in rats did not show any toxicity signs or mortality at the fixed dose of 2000 mg/kg body weight during the observation periods. Therefore, the extracts may be safe, and the oral LD_50_ (the dose of the extract that causes the death of 50% of animals in the test groups) is considered greater than 2000 mg/kg in rats.

#### 3.5.2. Effect of *Psychotria densinervia* Hydroethanolic Leaf and Bark Extracts on Foods and Water Consumption

**Figure 4:**
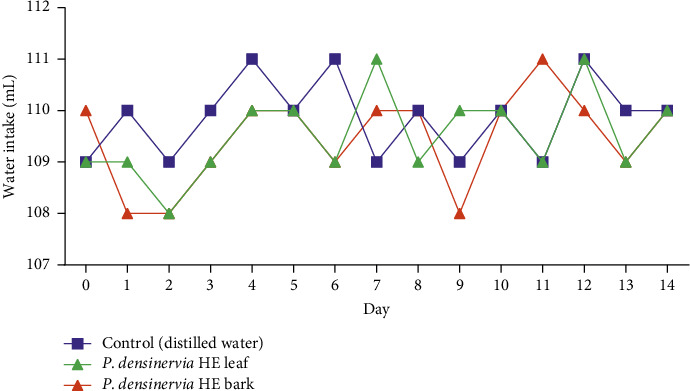
Effects of acute oral administration of *Psychotria densinervia* hydroethanolic leaf and bark extracts on water intake of Wistar rats with a dose of 2000 mg/kg. *P. densinervia* HE = *Psychotria densinervia* hydroethanolic; each values represent the quantity of water intake in ml and are expressed as mean ± SD; *n* = 5.

**Figure 5:**
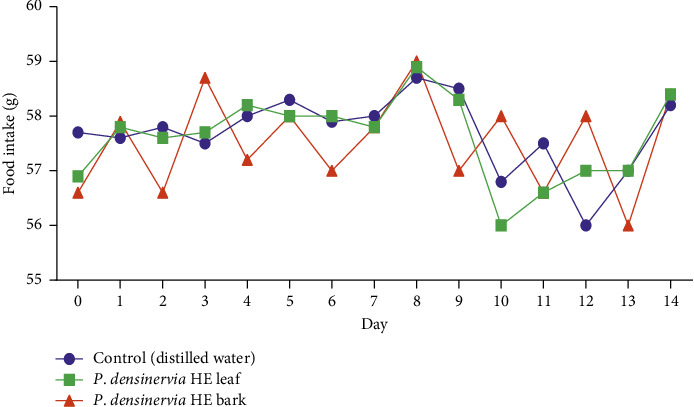
Effects of acute oral administration of *Psychotria densinervia* hydroethanolic leaf extract on food intake of Wistar rats with a dose of 2000 mg/kg. *P. densinervia* HE = *Psychotria densinervia* hydroethanolic; values represent the quantity of food intake in each group in g and are expressed as mean ± SD; *n* = 5.

During the experimentation period (14 days), there was no significant (*p* > 0.05) change in water (Figure 4) or food intake (Figure 5) between *P. densinervia* hydroethanolic leaf extract, P. hydroethanolic bark extract, and the control group (distilled water).

#### 3.5.3. Effect of *Psychotria densinervia* Hydroethanolic Leaf and Bark Extracts on Rats' Body Weight

**Figure 6:**
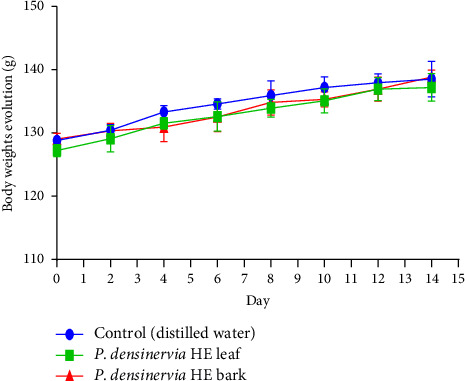
Effects of acute oral administration of *Psychotria densinervia* hydroethanolic leaf and bark extracts on Wistar rats' body weights with a dose of 2000 mg/kg. *P. densinervia* HE = *Psychotria densinervia* hydroethanolic; values represent the evolution of the rats body weights in each group in g and are expressed as mean ± SD; *n* = 5.

The body weight of the control group as well as those administered *Psychotria densinervia* hydroethanolic leaf and bark extracts increased progressively throughout the study period, as shown in Figure 6. There were no significant variations (*p* > 0.05) between the three groups. Thus, *P. densinervia* hydroethanolic leaf and bark extracts did not acutely affect body weight at a dose of 2000 mg/kg in rats.

### 3.6. UPLC-ESI/MS Analysis

**Figure 7:**
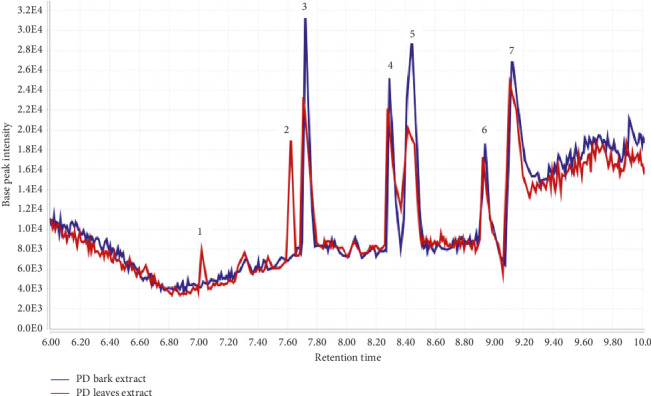
*P. densinervia* hydroethanolic extracts of bark (PD bark extract) and leaf (PD leaf extract) total ion chromatograms.

**Figure 8:**
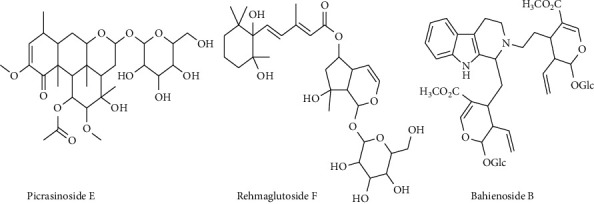
Structure of annotated compounds 1, 2, and 5 from *P. densinervia* total ion chromatograms.

Chromatographic profiling of the hydroethanolic extracts of *P. densinervia* leaf and bark led to the annotation of 5 out of the 7 major peaks (Table 4) from the obtained total ion chromatograms (Figure 7). Among these 7 compounds, 5 are identified as alkaloids (compound 3, 4, 5, 6, and 7) and 2 as terpenoids. 6-amino-2-(decanoylamino)-3-(2,3-dihydroxypropoxy) hexanoic acid (**3**), 1-N-[2-[2-(dimethylamino) ethoxy] ethyl]-4-[2-[2-(dimethylamino) ethyl-methylamino]ethoxy]-1-N-methylhexane-1,3,6-triamine (**4**), Bahienoside B (**5**), and the unannotated compounds **(6)** and **(7)** were found in both hydroethanolic leaf and bark extracts while picrasinoside E (**1**) and rehmaglutoside F (**2**) were found only in the hydroethanolic leaves extract.  Figure 8 shows the structure of annotated compounds 1, 2, and 5 from *P. densinervia* total ion chromatograms.

## 4. Discussion

In recent decades, the primary focus on controlling obesity has been on preadipocyte differentiation and proliferation suppression. Previous research has used the human liposarcoma SW-872 cell line as an adipocyte cell model. These cells possess a physiological reaction that is analogous to that of mature adipocytes. These cells have an advantage over mouse 3T3-L1 adipocytes because they are derived from humans and do not need an incubation cocktail for proliferation. The SW-872 cell line constitutively expresses PPAR*γ* and C/EBP*α*, which are essential for adipocyte formation [27]. Using the SW-872 cell line, the cytotoxicity assay of *P. densinervia* hydroethanolic leaf and bark extracts at various concentrations revealed that treated cells exhibited no signs of cellular toxicity. When *P. densinervia* hydroethanolic leaf and bark extracts were used, higher percentages of viability were achieved. No change in cell morphology or appearance was observed.

The *P. densinervia* hydroethanolic leaf and bark extracts were tested at concentrations of 25, 50, 100, and 200 μg/mL for their antiadipogenic properties. The *P. densinervia* hydroethanolic leaf extract showed more antiadipogenic action than the bark extract at the tested concentrations. Our previous study exhibited that *P. densinervia* hydroethanolic leaf extract content more showed phenolic compounds (270.05 ± 7.53 mg catechin equivalent per gram of extract) than the bark extract (138.89 ± 0.91 mg catechin equivalent per gram of extract) [19]. The present UPLC-ESI/MS analysis exhibited that alkaloids 6-amino-2-(decanoylamino)-3-(2,3-dihydroxypropoxy) hexanoic acid (**3**); 1-N-[2-[2-(dimethylamino) ethoxy] ethyl]-4-[2-[2-(dimethylamino) ethyl-methylamino] ethyl]-1-N-methylhexane-1,3,6-triamine (**4**), bahienoside B (**5**), and unannotated compounds 6 and 7 are identified as major compounds in both leaf and bark extracts. In addition to that alkaloids, 2 terpenoids (picrasinoside E (**1**) and rehmaglutoside F (**2**)) have also been identified in the leaf extract. Adipogenic transcription factors such as C/EBP*α* and PPAR-*γ* are known to activate and express genes specific to adipocytes, such as fatty acid synthase, fatty acid binding protein, leptin, adiponectin, and others [28, 29]. Phenolic substances have been shown to reduce C/EBP*α* and PPAR-*γ* and prevent lipid formation in adipocytes [30]. Although the activity of the identified alkaloids and terpenoids in this study has never been evaluated against adipogenesis, several studies have reported the antiadipogenic effect of alkaloids and terpenoids through the downregulation of C/EBP*α* and PPAR-*γ* [31–35].

Ox-LDLs are involved in the activation of adipogenic transcription factors [36] and increase proinflammatory cytokine production, and cell proliferation and differentiation [37, 38], resulting in the development of obesity. High ox-LDL binds and internalizes many scavenger receptors, including CD36, SR-BI, LOX-1, and SRA [38, 39]. Ox-LDL levels have also been linked with a decrease in adiponectin and HDL cholesterol levels with a great increase in triglyceride levels; this is the primary process by which obesity develops [15, 16]. In this study, LDL was oxidized in vitro by copper sulfate (CuSO_4_). According to Rahman et al. [40], the oxidation of LDL molecules and their subsequent molecular rearrangement into conjugated dienes were caused by the Cu^2+^ ions supplied by CuSO_4_. The increase in oxidation lag times, time 50, and TBARSs formation inhibition percentages by *P. densinervia* hydroethanolic leaf and bark extracts was obtained in a concentration-dependent manner. An increase in lag times and time 50 indicates that the antioxidant agents are inhibiting LDL oxidation [40]. TBARS is an indicator to measure the level of lipid oxidation, and the increase in its inhibition percentages in this study reveals the inhibition of LDL oxidation by *P. densinervia* hydroethanolic leaf and bark extracts. The leaf extract exhibited the highest lag times, time 50, and highest percentages of TBARS inhibition compared to the bark extract. Our previous result indicated that *P. densinervia* hydroethanolic leaf extract contained more flavonoids (23.43 ± 0.03 mg catechin equivalent per gram of extract) [19] than the bark extract (20.06 ± 0.032 mg catechin equivalent per gram of extract), and these flavonoids might be able to bind to the LDL molecule protect them against oxidation by trapping free radicals and chelate metal ions [41]. Several studies exhibited the properties of alkaloids and terpenoids against the oxidation of LDL [42–47]. Thus, the obtained activities in these studies might be also due to the presence of the identified alkaloids and terpenoids.

Earlier studies had reported a positive and significant correlation between adipogenesis and circulating levels of ox-LDL [12–14]. According to Holvoet, De Keyzer, and Jacobs [48], ox-LDL increases triglyceride production by inducing the expression of lipoprotein lipase and by inducing the accumulation of fatty acids in adipocytes. In the other hand, many studies have demonstrated that ox-LDLs were shown to bind and be internalized by many scavenger receptors in 3T3-L1 cells adipocytes, causing the upregulation of preadipocyte factor-1, which are the key of adipocyte differentiation [49, 50]. The uptake of ox-LDL in 3T3-L1 adipocytes also triggers aberrant ROS-mediated plasminogen activator inhibitor-1 expression, which may be involved in the adipogenesis [51]. Thus, the inhibition of LDL oxidation may play an important role in the management of adipogenesis.

Rats in the current investigation exhibited no signs of toxicity after the oral acute administration of a single dose of 2000 mg/kg. Therefore, the LD_50_ was greater than 2000 mg/kg and may not be considered toxic. Compounds having an LD_50_ between 2000 and 5000 mg/kg are classified as unclassified or in category 5 by the OECD under its globally harmonized classification system (GHS) for chemical compounds and combinations that are considered nontoxic [24]. As stated by Ugwah-Oguejiofor et al. [52], variations in the food, water intake, and body weight of experimental animals have been used as indications of their health status. Appetite controls the body's need for food, which is crucial for maintaining a healthy weight. Animals' physiological and pathological states are indicated by their body weights [53, 54]. In this study, *P. densinervia* hydroethanolic leaf and bark extracts did not affect the consumption of food or water. Nonetheless, the body weights of the control and treated animals increased gradually and normally. The safety of *P. densinervia* hydroethanolic leaf and bark extracts in rats at the tested dose (2000 mg/kg) is thus supported by this finding.

## 5. Conclusion

This study revealed that *P. densinervia* hydroethanolic leaf and bark extracts possess antiadipogenic activity by increasing the percentage of lipid uptake inhibition and decreasing the relative lipid content into SW872 adipocytes. *P. densinervia* hydroethanolic leaf and bark extracts also inhibited the oxidation of LDL through the increase of lag times and the increase of TBARS percentages inhibition. The UPLC-ESI/MS analysis showed the presence of alkaloids, as major compounds in both leaf and bark extracts. In addition, terpenoids have also been identified in *P. densinervia* hydroethanolic leaf extract. During this study, the leaf extract was more potent than the bark extract. The hydroethanolic leaf and bark extracts did not exhibited any sign of cytotoxicity in SW-872 cells. Higher percentages of cells viability were achieved. No change in cell morphology or appearance was observed. Wistar albino rats in the oral acute toxicity test did not exhibited any signs of toxicity after the administration of a single dose of 2000 mg/kg of the hydroethanolic leaf and bark extracts. Therefore, the LD50 was greater than 2000 mg/kg. Thus, our finding supports the safety of *P. densinervia* hydroethanolic leaf and bark extracts in rats at this tested dose (2000 mg/kg).

## Figures and Tables

**Table 1:** IC50 of *Psychotria densinervia* hydroethanolic leaf and bark extracts on lipid accumulation inhibition in SW-872 adipocytes.

	Leaf extract	Bark extract	Orlistat
IC_50_ (μg/mL)	41.47 ± 0.50	107.50 ± 0.90^###^	38.45 ± 1.70

^###^ *p* < 0.001: significant difference compared to orlistat.

**Table 2:** Inhibition percentage of the formation of thiobarbituric acid reactant substances (TBARS) and IC_50_ values of *Psychotria densinervia* hydroethanolic leaf and bark extract.

	Concentration (mg/mL)	Percentage inhibition (%)	IC_50_ (mg/mL)
*P.densinervia* HE leaf extract	0	0.0 ± 0.0	0.20 ± 8.50
	0.25	57.2 ± 0.4⁣^∗∗∗^	
	0.5	66.8 ± 0.8⁣^∗∗^	
	1	94.5 ± 0.8	

*P.densinervia* HE bark extract	0	0.0 ± 0.0	0.24 ± 6.50
	0.25	38.5 ± 1.0⁣^∗∗∗^	
	0.5	53.2 ± 0.8⁣^∗∗∗^	
	1	72.7 ± 0.8⁣^∗∗∗^	

Quercetin	0	0.0 ± 0.0	0.10 ± 5.10
	0.25	74.0 ± 0.6	
	0.5	79.9 ± 0.6	
	1	95.6 ± 0.4	

*Note:* Values are expressed as mean ± SD; *n* = 3.

Abbreviation: *P. densinervia* HE = *Psychotria densinervia* hydroethanolic.

⁣^∗∗^*p* < 0.01.

⁣^∗∗∗^*p* < 0.001: significant differences compared to quercetin.

**Table 3:** Pearson correlation between LDL oxidation inhibition versus percentage of lipid uptake inhibition and relative lipid content.

	Lipid uptake inhibition percentage	Relative lipid content	Lipid uptake inhibition percentage
LDL oxidation inhibition	0.89⁣^∗^	−0.89⁣^∗^	
Relative lipid content			−1⁣^∗∗∗^

⁣^∗^Correlation significant at 0.05 level.

⁣^∗∗∗^Correlation significant at 0.001 level.

**Table 4:** Annotated compounds from the total ion chromatograms of *P. densinervia*.

	Rt	Acquired m/z	Adduct	Exact mass	Formula	Annotated compounds	Extracts
1	7.07	637.2990	[M + Na]^+^	637.2836	C_30_H_46_O_13_	Picrasinoside E (**1**)	PDL
2	7.62	621.2888	[M + Na]^+^	621.2887	C_30_H_46_O_12_	Rehmaglutoside F (**2**)	PDL
3	7.71	391.2793	[M + H]^+^	391.2808	C_19_H_38_N_2_O_6_	6-Amino-2-(decanoylamino)-3-(2,3-dihydroxypropoxy)hexanoic acid (**3**)	PDL PDB
4	8.29	427.3721	[M + Na]^+^	427.3736	C_20_H_48_N_6_O_2_	1-N-[2-[2-(dimethylamino)ethoxy]ethyl]-4-[2-[2-(dimethylamino)ethyl-methylamino]ethoxy]-1-N-methylhexane-1,3,6-triamine (**4**)	PDL PDB
5	8.41	925.6194	[M + Na]^+^	925.3582	C_44_H_58_N_2_O_18_	Bahienoside B (**5**)	PDL PDB
6	8.93	547.3888	[M + H]^+^	547.3899	C_35_H_50_N_2_O_3_	Unannotated (**6**)	PDL PDB
7	9.12	161.0680	nd	nd	Nd	Unannotated (**7**)	PDL PDB

## Data Availability

We have the data of this research article and can provide it as per the request.

## Appendix A: Limitations of the Study and Future Direction

The study has been carried out using only in vitro antiadipogenic models. Therefore, in the future, the in vivo antiobesity activity will be evaluated, including the PPAR gamma gene expression assays, in order to offer a better approach to understanding the molecular mechanism underlying the antiobesity effects of *Psychotria densinervia* hydroethanolic extracts. In addition, the subacute toxicity study will be evaluated, in which morphological and biochemical measurements will be made on different organs to support the safety of the extracts.
